# Efficacy assessment of commercially available natural products and antibiotics, commonly used for mitigation of pathogenic *Vibrio* outbreaks in Ecuadorian *Penaeus (Litopenaeus) vannamei* hatcheries

**DOI:** 10.1371/journal.pone.0210478

**Published:** 2019-01-30

**Authors:** María Auxiliadora Sotomayor, Jessica Karina Reyes, Leda Restrepo, Cristóbal Domínguez-Borbor, Martha Maldonado, Bonny Bayot

**Affiliations:** 1 Centro Nacional de Acuicultura e Investigaciones Marinas, CENAIM. Escuela Superior Politécnica del Litoral, ESPOL, Guayaquil, Ecuador; 2 Universidad Estatal Península de Santa Elena, UPSE, Facultad de Ciencias del Mar, FCM, Vía Santa Elena-La Libertad, La Libertad, Ecuador; 3 Facultad de Ingeniería Marítima, Ciencias Biológicas, Oceánicas y Recursos Naturales, FIMCBOR, Escuela Superior Politécnica del Litoral, ESPOL, Guayaquil, Ecuador; Hellenic Center for Marine Research, GREECE

## Abstract

Bacterial diseases cause high mortality in *Penaeus (Litopenaeus) vannamei* postlarvae. Therefore, appropriate application of efficient therapeutic products is of vital importance for disease control. This study evaluated through *in vitro* analyses the antimicrobial effectiveness of commercial therapeutic products used for *P*. *vannamei* bacterial diseases and antibiotics against pathogenic *Vibrio* strains circulating in Ecuadorian hatcheries. Twenty strains were isolated from 31 larvae samples with high bacterial counts from 10 hatcheries collected during mortality events. The strains virulence was verified through challenge tests with *Artemia franciscana* nauplii and *P*. *vannamei* postlarvae. Through 16S rRNA sequence analysis, strains showed a great similarity to the *Vibrio* sequences reported as pathogens, with 95% belonging to the *Harveyi* clade. Through antibiograms and minimal inhibitory concentration (MIC) *in vitro* tests we found that furazolidone, ciprofloxacin, chloramphenicol, norfloxacin, nalidixic acid, florfenicol, fosfomycin and enrofloxacin inhibited the growth of all or most of the strains. Less efficient antibiotics were penicillin, oxytetracycline and tetracycline. A multiple antibiotic resistance (MAR) index of 0.23 showed some level of resistance to antibiotics, with two MAR prevalent patterns (Penicillin-Oxytetracycline and Penicillin-Oxytetracycline-Tetracycline). From a total of 16 natural products (five probiotics, nine organic acids and two essential oils), only three (one probiotic, one organic acid and one essential oil) were effective to control most of the strains. Shrimp producers can apply relatively simple *in vitro* analyses, such as those employed in this study, to help take adequate management decisions to reduce the impact of bacterial diseases and increase profit.

## Introduction

The high demand of postlarvae to support the cultured shrimp industry and consequently the intensification at hatchery level, together with the trade of aquatic animals and their associated products, has increased the occurrence of infectious pathogens in this production stage [1]. One of the main concerns in shrimp hatcheries are the bacterial pathogens [2]. *Vibrio* spp, such as *Vibrio harveyi* [3–8], *Vibrio alginolyticus* [9, 10] and *Vibrio campbellii* [7, 11, 12] are recurrent pathogens in shrimp hatcheries in America and Asia. In Ecuador, shrimp hatcheries have suffered from some bacterial diseases caused by pathogens of the *Vibrio* genus, such as Bolitas nigricans syndrome, caused by *V*. *harveyi* [3], and Zoea 2 syndrome, caused by *V*. *harveyi* and *V*. *alginolyticus* [4]. Therefore, the efficiency of therapeutic products is of vital importance for the control of aquaculture diseases.

Antibiotics are extensively used as prophylactics against bacterial pathogens [13]. However, the use of antibiotics carries important disadvantages, these being residues in aquaculture products [14–16], development and propagation of resistance between pathogens, including human pathogens [17]. For these reasons, the regulation of antibiotics is rigorously controlled, resulting in few antibiotics authorized for use in aquaculture. In this context, alternative strategies of disease control are necessary to replace antibiotics for use in animal production, which has led to consider the use of natural products to control the growth of pathogens in shrimp hatcheries. The administration of probiotics is one of the alternative strategies that may be used in aquaculture [13]; their benefits include the potential for colonization in the gastrointestinal tract, selective antagonism against bacterial pathogens, improvement of the shrimp immune system, enhanced shrimp growth and survival, degradation of detritus and maintenance of water quality [18–20]. The use of organic acids, produced by organisms and used as preservatives and bacterial control in food, agriculture, and animal production, is another potential strategy to control bacterial diseases in animal production [21–23]. They inhibit the growth of pathogenic *V*. *harveyi*, *Vibrio cholera*, *V*. *alginolyticus*, *Vibrio parahaemolyticus* and *V*. *campbellii* [24–26], exhibit immunostimulant properties [24, 27], and improve the nutritional and health state of shrimp [28, 29]. Similarly, essential oils have shown to have antimicrobial [30], antioxidant [31] and antifungal [32] properties, which can be an alternative to the use of additives and drugs in shrimp production [33]. Although the use of organic acids and essential oils in Ecuadorian hatcheries is relatively new, there is an increasing demand for their application as control strategies of bacterial diseases in shrimp hatcheries. In general, there are a huge number of products marketed as therapeutic products for shrimp hatcheries worldwide, therefore producers should take suitable decisions as to which products are effective based on technical information and further tests in their own facilities.

The main objective of this study was to determine through *in vitro* analyses the antimicrobial effectiveness of antibiotics and some commercial products used in Ecuador as therapeutic agents for shrimp larviculture. To determine this, we first performed a survey to identify the pathogenic bacterial strains circulating in Ecuadorian shrimp hatcheries, confirming their virulence through challenge tests and verifying their molecular similarity with previously reported pathogenic *Vibrio*. Then, we tested the antimicrobial effectivity of some antibiotics and commercial products used in Ecuador as therapeutics against the pathogenic circulating strains through *in vitro* tests.

## Material and methods

### Sample collection and processing

In 2015, 31 samples of *Penaeus (Litopenaeus) vannamei* larvae [from Nauplii 5 (N5) to 13 days postlarvae (PL13)] were collected from tanks of 10 shrimp hatcheries (Santa Elena, Ecuador) during mortality events. Samples were sent by farmers to the Centro Nacional de Acuicultura e Investigaciones Marinas (CENAIM, Santa Elena, Ecuador) for the quantification of shrimp bacterial load (microbiologic analysis services performed by CENAIM). Bacterial strains isolated from these samples were used for this study. Larvae presented clinical signs of abnormal swimming behavior, empty digestive tract, low activity and retardation of larval development. Samples were transported to the research facilities of CENAIM taking a maximum time of two hours from sampling at the hatcheries to processing at the laboratory. At the laboratory, they were rinsed with 2% sterile NaCl solution and each sample (1 g of larvae) was macerated to homogenize the bacterial load associated with the animals.

### Isolation and preservation of bacterial strains

Aliquots (100 μL) of serial 10-fold dilutions (10^−3^ to 10^−5^) of larvae were macerated in duplicate in 2% sterile NaCl solution and plated on marine agar 2216 (MA, Difco). The same procedure was performed with serial 10-fold dilutions (10^−1^ to 10^−3^) on thiosulfate citrate bile salt sucrose agar (TCBS, Difco). All plates were incubated at 30°C. After one to two days of growth, bacterial counts were performed from plates containing 30 to 300 colonies. Bacterial counts were expressed as colony-forming units (CFU) per gram. Presumptive pathogenic strains were selected from colonies of samples with high bacterial counts on MA (>10^6^ CFU g^-1^) or TCBS (>10^5^ CFU g^-1^) agars. The criteria of selection were: (1) all different bacterial strains by morphological criterion and (2) luminescent strains. All selected strains were coded and frozen at -80°C after addition of trypticase soy broth (TSB, Difco) supplemented with 2.0% (w/v) NaCl and 20% (v/v) glycerol.

### Challenge tests

The pathogenicity of the presumptive pathogenic strains was first evaluated in brine shrimp *Artemia franciscana* nauplii, following the procedures described by [34], with few modifications. Briefly, 1 g cysts of *A*. *franciscana* (Batch 02143, INVE Aquaculture, Belgium) were hydrated under continuous aeration in 100 mL of filtered and autoclaved distilled water for 1 h, and then transferred to a mixture of 10 mL of sodium hypochlorite (10%) and 15 mL of sodium hydroxide (40%), until the cysts changed color from brown to orange. The decapsulated cysts were washed with filtered and autoclaved sea water and transferred to an imhoff funnel with 500 mL of filtered and autoclaved sea water, providing continuous aeration and illumination with a white lamp, remaining at 28°C in a sterile environment for 24 h. Nauplii were harvested under sterile conditions with an autoclaved mesh of 100 μm. Eighty-four groups, each composed of 30 *Artemi*a nauplii, were transferred to 50 mL sterile tubes containing 30 mL of filtered and autoclaved sea water. Eighty tubes were used to test the pathogenicity of the 20 bacterial isolates (four replicates per strain) and four tubes were used for the negative control (without bacteria, four replicates). Bacteria were activated on trypticase soy agar (TSA, Difco) and a colony from each strain was transferred to 150 mL of TSB and incubated for seven hours at 30°C. Bacterial cells, at a density of 10^6^ cell mL^-1^, were added to the corresponding sterile tubes immediately after the transfer of the *Artemia* nauplii. Nauplii from all treatments and control were fed once with an inactivated *V*. *alginolyticus* commercial probiotic (10^7^) [35] four hours after infection. For this, the probiotic was cultured in liquid medium for six hours, inactivated by heat (autoclaved at 121°C for 20 min), centrifuged at 10000 rpm for 10 min and resuspended with autoclaved seawater. Water exchange (50%) was performed 24 hours after infection and the mortality of *Artemia* was quantified 48 h post-infection. The whole challenge was performed in a Class II Biological Security Cabinet (CSB-180 A). To verify the asepsis condition of the negative control, water samples were collected at the end of the challenge, thus verifying the absence of *Vibrio* growth in TCBS agar.

Bacterial strains causing higher mortalities in the *Artemia* challenge test were selected and their pathogenicity was again verified by a challenge test using healthy *Penaeus vannamei* postlarvae, following the procedures described by [36]. Bacteria were activated following the procedure described in the *Artemia* challenge test. Thirty shrimp postlarvae (PL2) per replicate were distributed in sterile petri dishes and exposed to the corresponding bacterial treatment, at a concentration of 10^8^ bacteria mL^-1^ for 6 min. Larvae were then transferred to 500 mL plastic containers containing 300 mL of sterile seawater and 10^6^ bacteria mL^-1^. Larvae were fed during the challenge with a pure culture of *Thalassiosira weissflogii* (913 cell mL^-1^) every 2 h after exposure to the bacteria. Survival was determined by counting the larvae every 4 h until 38 hours after infection. A negative control was also included in the challenge test (shrimp postlarvae PL2 without bacterial treatment). All treatments including the control had four replicates. The whole challenge was performed in a Class II Biological Security Cabinet (CSB-180 A), with constant aeration. To verify the asepsis condition of the negative control, water samples were collected at the end of the challenge, verifying the absence of *Vibrio* growth in TCBS agar.

### Bacterial characterization by 16S rRNA sequence analysis

Identification of the presumptive pathogenic strains was performed by 16S rRNA sequence analysis. Total genomic DNA was extracted from pure cultures of the bacterial strains after they were cultured on TSA and incubated for 24 h at 30°C. Bacteria were lysed by incubation at 55°C for 1 h in 200 μL of STE buffer (10 mM Tris-HCl, 1 mM EDTA and 100 mM NaCl, pH 8), followed by purification, adding an equal volume of phenol-chloroform-isoamylalcohol (25:24:1) and continuing with a chloroform-isoamylalcohol extraction (24:1). DNA was recovered by adding ethanol (100%) followed by centrifugation at 13000 rpm for 10 min. The pellet was washed with 70% ethanol, dried and resuspended in 50 μL of ultrapure water (pH 7.0). DNA was preserved at -20°C for further use. DNA concentration and purity were estimated with a Varioskan LUX multimode microplate reader (Thermo Fisher Scientific). The 16S rRNA complete gene was amplified using primers suggested by [37] (27F: 5'-AGAGTTTGATCMTGGCTCAG-3', 1492R: 5'-TACGGYTACCTTGTTACGACTT-3'). PCR was performed in a 30 μL reaction mixture containing 1X Buffer NH_4_ (Bioline, Sydney, Australia), 2.5 mM MgCl_2_ (Invitrogen, Carlsbad, CA), 2 mM of each dNTP, 0.3 μM of each primer, 0.5 units of Taq DNA polymerase and 2 μL of DNA. PCR cycling conditions were: DNA denatured for 5 min at 94°C, 35 cycles at 94°C for denaturation, 1 min at 52°C for annealing step and 1 min at 72°C for elongation; and a last cycle of 10 min at 72°C to complete the elongation. Amplicons were separated by 1.5% (w/v) agarose gel electrophoresis, stained with SYBR Safe DNA gel stain (Thermo Fisher Scientific), and illuminated under UV light. Images were captured with an E-Gel Imager System (Thermo Fisher Scientific). PCR products were purified and dissolved in 30 μL of ultrapure water for direct sequencing (Macrogen, Korea). BigDye Terminator Cycle sequencing kit (Perkin Elmer) was used for the sequencing. The sequencing products were analyzed with the ABI 3000 sequencer (Applied Biosystems, Foster City, CA, USA). A phylogenetic analysis was carried out with the complete 16S rRNA sequences (1465 bp) from the bacterial isolates, together with 16S rRNA sequences (n = 362) of different *Vibrio* (pathogens and non-pathogens) obtained from GenBank. The amino acid sequence alignments were generated with ClustalW and the specific region of 16S rRNA was identified using Bioedit 7.0.0. [38]. Phylogenetic trees were built using Maximum Parsimony (MP), Maximum Likelihood (ML), Neighbor Joining (NJ) and Bayesian Inference (BI). The molecular evolution model was selected through a wide range of phylogenetic and evolutionary tools using a dataset composed only of the unique haplotypes and sequences obtained in the present investigation. JModeltest 2.0 was used to test the evolution models based on the hierarchical likelihood ratio test [39]. Values of amino acid substitutions per site for the gene were calculated with MEGA 6.0 (Molecular Evolutionary Genetics Analysis). The 16S rRNA sequences were deposited in GenBank under accession numbers MH997724 to MH997742.

### Antimicrobial effectiveness

The antimicrobial effectivity of 16 natural products (five probiotics, nine organic acids and two essential oils, Table 1) used in Ecuador as therapeutic agents against shrimp bacterial diseases and eleven antibiotics was screened in terms of the susceptibility of the pathogenic circulating strains to these products through antibiogram and minimal inhibitory concentration tests (Table 1). We denominated as pathogenic circulating strains those strains that caused high mortality (> 50%) in the *Artemia* and shrimp postlarvae challenge tests and presented molecular similarity to species previously reported as *Vibrio* pathogens. The product details are provided in S1 Table.

**Table 1:** Description of the products marketed in Ecuador as therapeutic agents against shrimp bacterial disease.

Product code	Declared composition	Declared dosage / dosage used by producers	Presentation	Country manufacturer
**P1**	Probiotic microorganisms: total aerobes^a^. Concentration: > 4 × 10^9^ CFU g^-1^	2–10 μg mL^-1^	Powder	USA
**P2**	Probiotic microorganisms: total aerobes^a^. Concentration: ≥ 2 × 10^9^ CFU g^-1^	5 μg mL^-1^	Powder	USA
**P3**	Strains of *Bacillus subtilis*, *Bacillus licheniformis* and *Bacillus pumilus*. Concentration: minimum 2 × 10^10^ CFU g^-1^	1 to 5 g kg^-1^	Powder	USA
**P4**	Mixture of strains of *Bacillus* spp. Concentration: 5 × 10^10^ CFU g^-1^	100–200 g ha^-1^	Powder	USA
**P5**	*Vibrio alginolyticus*. Concentration: 1 × 10^8^ CFU mL^-1^	10 mL t^-1^	Liquid	Ecuador
**OA1**	Calcium formate, calcium propionate, premix carvacrol and thymol, premix allicin, yeast cell wall, calcium lactate, nucleotide, vitamin C 35%, fumaric acid, calcium citrate, organic zinc, organic manganese, inositol, vitamin E, sodium acetate, benzoic acid, BHT, betaglucans, organic iron, citric acid, niacin, vitamin A, zinc, calcium pantothenate, vitamin B6, potassium sorbate, magnesium chloride, copper sulfate, organic copper, monosodium phosphate, vitamin D3, organic selenium and sodium selenium	1–7 kg t^-1^ of feed	Powder	Ecuador
**OA2**	Formic acid, propionic acid, ammonium formate, acetic acid, silic acid and vermiculite	0.6 kg t^-1^ of feed	Powder	Austria
**OA3**	Calcium propionate 16%, calcium formate 18% and calcium carbonate 66%	1–2 kg t^-1^	Powder	Ecuador
**OA4**	Propionic acid 25%, formic acid 25% and formaldehyde 15%	1–3 kg ha^-1^	Powder	Ecuador
**OA5**	Formic acid, and its salts, mixture of flavors (essences and plant extracts: *Allium sativum*, *Origanum vulgare*, *Cinnamomum zeylanicum*, *Eugenia caryophyllata*), propionic acid and its salts, citric acid, malic acid, anti-caking agent	2–3 kg t ^-1^ of feed	Powder	Spain
**OA6**	Lactic acid 23%, fumaric acid 20%, citric acid 20%, malic acid 25% and succinic acid 10%	2–4 μg mL^-1^	Powder	Ecuador
**OA7**	Acid formic 35.4%, formate 34.6% and potassium 30.0%	2–5 kg t ^-1^ of feed	Powder	Germany
**OA8**	Formaldehyde 35%: 28.6%, propionic acid 10%, bentonite 39% and silicic acid 22.4%	1 kg t ^-1^ of feed	Powder	Spain
**OA9**	Mixture of short chain organic acids, acetic acid, propionic acid, formic acid and formaldehyde	0.5–2 kg t^-1^ of feed	Powder	Spain
**EO1**	Oregano oil extract	1–5 mL t^-1^	Liquid	USA
**EO2**	Highly concentrated mix of essential oils	1–10 mL t^-1^	Liquid	Spain

Probiotics: P1, P2, P3, P4 and P5; Organic acids: OA1, OA2, OA3, OA4, OA5, OA6, OA7, OA8 and OA9; Essential oils: EO1 and EO2.

^a^ Specific bacterial strain are not declared in the product

### Susceptibility of pathogenic *Vibrio* strains to antibiotics

The susceptibility of the pathogenic circulating strains to antibiotics was determined by the *in vitro* agar diffusion method [40, 41] using antimicrobial susceptibility test discs (diameter 6 mm, Bioanalyse and Oxoid). For this study, eleven different antimicrobial discs were used per duplicate [chloramphenicol (C, 30 μg), ciprofloxacin (CIP, 5 μg), fosfomycin (FF, 10 μg), furazolidone (FUR, 100 μg), norfloxacin (NOR, 10 μg), oxytetracycline (T, 30 μg), penicillin (P, 10 U), tetracycline (TE, 30 μg), nalidixic acid (NAL, 30 μg), enrofloxacin (E, 5 μg) and florfenicol (F, 30 μg)]. Isolates were activated on TSA and incubated at 30°C for 24 h. Afterwards, a colony from each isolate was transferred to TSB liquid medium (2% NaCl) and incubated again at 30°C for 4 h. The bacterial suspensions were standardized with McFarland´s 0.5 Barium Sulfate Standard Solution (1.5 × 10^8^ CFU mL^-1^) and diluted to 10^6^. Standardized bacterial suspensions (100 μL) were inoculated onto Mueller-Hinton plates using sterile cotton buds. Plates were incubated agar down between 24 and 48 h at 30°C. Diameters of inhibition halos surrounding the discs were measured and expressed in millimeters. Results were interpreted as sensitive, intermediate or resistant, following the guidelines of the Clinical and Laboratory Standards Institute [42, 43].

The minimal inhibitory concentration (MIC) for the antibiotics approved for use in aquaculture (oxytetracycline and florfenicol, 99% purity, Zhejiang Medicines and Health Company, China) on the growth of the bacterial strains was also determined. The bacterial isolates were activated in TSB liquid medium and incubated at 30°C for 4 h. The bacterial suspensions were diluted with TSB liquid medium to an approximate density of 10^6^ CFU mL^-1^ by using McFarland´s 0.5 Barium Sulfate Standard Solution. Ten grams of each antibiotic were diluted in culture broth TSB with 2% NaCl solution. Sixteen concentrations of oxytetracycline, ranging from 1 to 3500 μg mL^-1^ were distributed into wells of round-bottom 96-well microplates. Each well was inoculated with 20 μl of bacterial suspension, including the positive control (bacterial growth at TSB culture, without any antibiotic). The microplates were incubated at 30°C between 24 and 48 h. All measurements were performed in triplicate, including those of the controls. Bacterial growth was detected by optical density at 620 nm (enzyme-linked immunosorbent assay ELISA reader, Varioskan Lux). MIC values were obtained as the lowest concentrations of each antibiotic that completely inhibited the bacterial growth. Absence of bacterial growth during the MIC process was confirmed on TSA agar. The same methodology was performed with the MIC tests for florfenicol, testing 15 concentrations of this antibiotic, ranging from 0.1 to 1000 μg mL^-1^. Finally, the patterns of multiple antibiotic resistance were analyzed to determine common patterns of resistance between the bacterial strains.

### Susceptibility of pathogenic *Vibrio* strains to probiotics

The susceptibility of the pathogenic circulating strains to five commercial shrimp probiotics (Table 1) was determined by the agar plug diffusion method [40]. Briefly, the probiotics were cultured on Mueller-Hinton agar at 30°C for 24 h (≈ 10^7^ CFU mL^-1^). In parallel, the pathogenic bacteria were cultured under the same conditions as the probiotics. After incubation, an agar-plot from the probiotic culture was aseptically cut and deposited on the agar surface of the plate inoculated with the pathogenic bacteria. Diameters of inhibition halos surrounding the agar-plots were measured and expressed in millimeters 24 and 48 h after the agar-plots were transferred. The strains were considered as: sensitive, intermediate and resistant when the diameters of the inhibition halos were ≥ 10 mm, between 4 and 9 mm and ≤ 3 mm, respectively.

### Susceptibilities of pathogenic *Vibrio* strains to organic acids and essential oils

The susceptibility of the pathogenic circulating strains to nine organic acids and two essential oils (Table 1) was determined in a similar way to that implemented for the MIC determination for antibiotics. The evaluated concentrations of organic acids and essential oils ranged between 100 to 3500 μg mL^-1^ and 100 to 3000 μg mL^-1^, respectively.

### Cell toxicity of selected products

The toxicity of the most efficient natural products, as well as the antibiotics authorized for use in aquaculture, was evaluated through the *in vitro* assay of cell viability of shrimp haemocytes [44]. Briefly, the assay was based on the ability of the mitochondria to convert yellow colored 3- (4,5-dimethylthiazol-2-yl) -2-5-diphenyltetrazole bromide (MTT) in purple colored formazan through the enzyme succinate dehydrogenase. The primary culture of shrimp haemocytes was activated in Hank’s salts for 75 min and exposed to each product for 2 h at different concentrations and then incubated for 2 h with 5 mg mL^-1^ of MTT in Hank’cs salts. The supernatant and the formazan crystals were diluted with isopropanol mixed with 0.04 N hydrochloric acid. The colorimetric reaction was read at 620 nm. The results were transformed to percent of cell viability, considering the primary culture of haemocytes without chemical exposure as the positive response of optimal cellular respiration (maximum cell viability). The concentrations evaluated of antibiotics were 100, 200, 400, 500, 1000, 2000, 2500, 3000, 4000, 5000, 6000 and 7000 μg mL^-1^. The concentrations evaluated for the natural products were below the corresponding minimum inhibitory concentrations.

### Data analysis

The index of multiple antibiotic resistance (MAR) was calculated according to [45] and calculated as the number of antibiotics to which the isolate was resistant divided by the total number of antibiotics tested.

Differences in cumulative mortality among treatments (presumptive pathogenic strains) were analyzed by one-way analysis of variance (ANOVA) at the end of each challenge test (48 and 38 h after infection for *A*. *franciscana* nauplli and *P*. *vannamei* postlarvae, respectively). The null hypothesis (no treatment effect) was rejected with a P-value ≤ 0.05. Variance homogeneity of all treatments was examined using the Bartlett test. Assumption of normality was examined through the Shapiro-Wilk normality test. Tukey’s Honest Significant Difference test was used to compare treatment means. The effect of differences between treatments was considered significant at P-value ≤ 0.05. The same statistical analysis was performed to evaluate differences of percent of cell viability (cell toxicity) for each one of selected products. All statistical tests were carried out with the R statistical software [46].

## Results

### Isolation and preservation of bacterial strains

A total of 20 different bacterial strains were selected by morphological and luminescence criteria from 121 colonies from 31 samples of *P*. *vannamei* larvae with high bacterial counts on MA (>10^6^ CFU g^-1^) or TCBS (>10^5^ CFU g^-1^) agars from 10 hatcheries collected during mortality events.

### Challenge tests

To estimate the virulence of the presumptive pathogenic strains, a first screening was performed challenging *A*. *franciscana* nauplii with all bacterial strains. All bacterial strains presented variable and high mortality in the challenge test (>74.2%, Table 2). A group of 11 strains caused the highest mortality in *A*. *franciscana* (> 95.0%, Table 2). The strain L15.19.1, belonging to this group caused 100% mortality in all replicates (Table 2). No significant mortality differences were observed among the *Artemi*a nauplii challenged with the remaining ten strains of this group (P > 0.995, Table 2). These strains were used for a second challenge test with shrimp *P*. *vannamei* postlarvae (PL2). No significant mortality differences were observed among the postlarvae challenged with these strains (P = 0.148, Table 2).

**Table 2:** Mortality (average ± standard deviation) of *Artemia franciscana* nauplii and *Penaeus vannamei* postlarvae (PL2) after 48 and 38 h of challenge, respectively, with presumptive pathogenic bacterial strains.

Bacterial strain	Mortality (%)	
	*A*. *franciscana* nauplii	*P*. *vannamei* postlarvae (PL2)	
L15.12.2	74.2 ± 5.7 ^a^	NI	
L15.13.2	77.5 ± 3.2 ^ab^	NI	
L15.31.1	79.2 ± 3.2 ^ab^	NI	
L15.29.2	80.8 ± 4.2 ^ab^	NI	
L15.23.3	83.3 ± 5.4 ^ab^	NI	
L15.26.1	83.3 ± 7.2 ^ab^	NI	
L15.26.3	85.0 ± 5.8 ^abc^	NI	
L15.5.2	87.5 ± 3.2 ^bd^	NI	
L15.19.2	87.5 ± 6.9 ^bd^	NI	
L15.25.3	95.0 ± 4.3 ^cde^	66.7 ± 13.0 ^a^	
L15.29.1	95.8 ± 4.2 ^cde^	64.2 ± 12.6 ^a^	
L15.23.1	96.7 ± 2.7 ^de^	61.7 ± 23.5 ^a^	
L15.25.1	96.7 ± 2.7 ^de^	72.5 ± 17.9 ^a^	
L15.21.1	97.5 ± 1.7 ^de^	NI	
L15.21.2	97.5 ± 3.2 ^de^	70.9 ± 18.3 ^a^	
L15.11.2	97.5 ± 3.2 ^de^	77.5 ± 3.2 ^a^	
L15.10.3	98.3 ± 1.9 ^de^	86.6 ± 22.5 ^a^	
L15.12.1	98.3 ± 3.3 ^de^	51.7 ± 10.4 ^a^	
L15.10.4	99.2 ± 1.7 ^e^	83.4 ± 4.7 ^a^	
L15.19.1	100.0 ± 0	71.7 ± 20.8 ^a^	
Negative control	0 ± 0	5.8 ± 9.6	

NI: strain not included in the challenge test. Means with different letters within a column indicate significant differences at P ≤ 0.05 by ANOVA and Tukey’s Honest Significant Difference tests.

### Bacterial characterization by 16S rRNA sequence analysis

**Fig 1:**
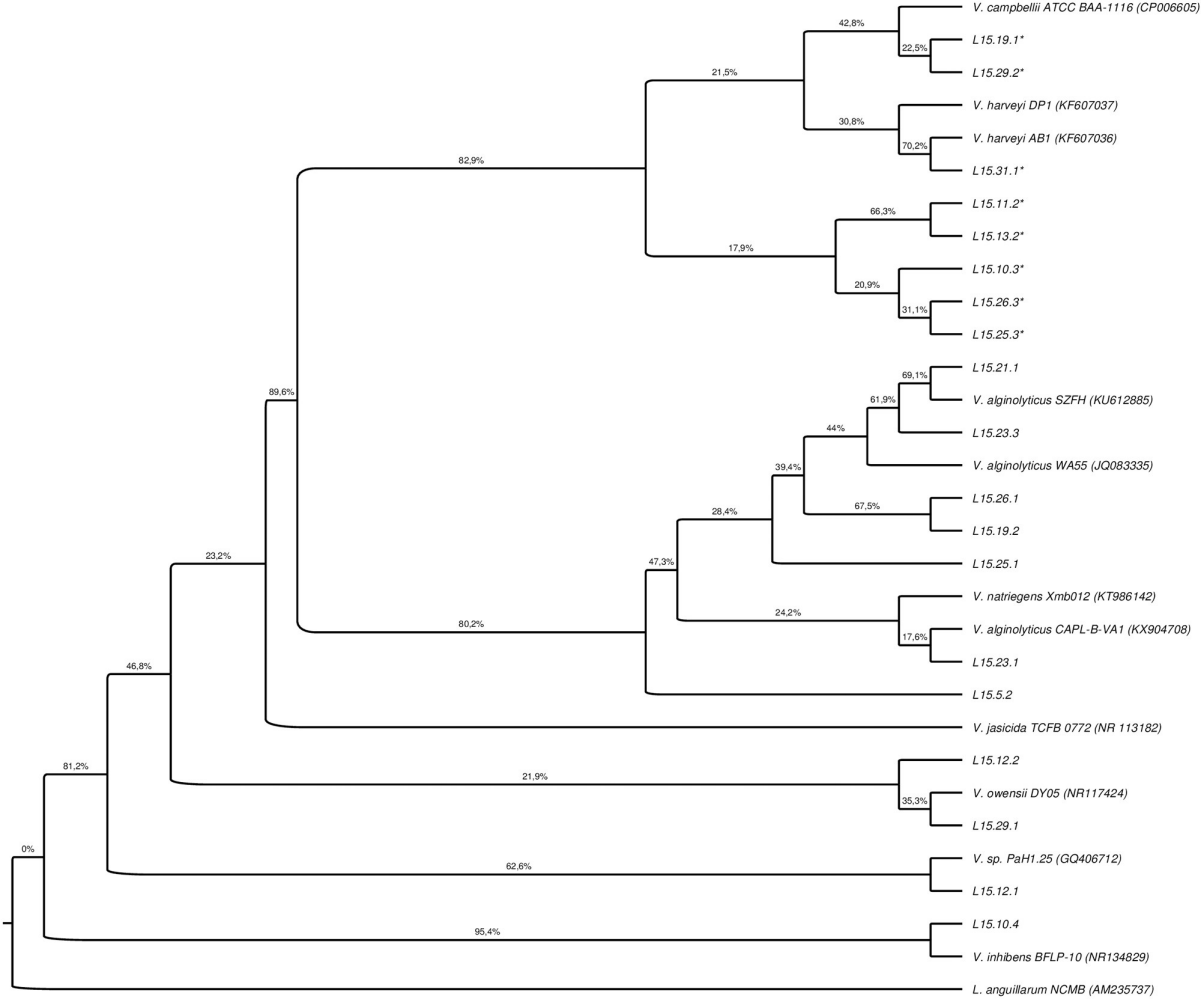
Phylogenetic tree of complete 16S rRNA sequences of the pathogenic circulating strains. The analysis included 11 related *Vibrio* pathogen sequences reported in GenBank. *Listonella anguillarum* was used as outgroup (GenBank accession no. AM235737). The evolutionary history was inferred by using the Maximum Likelihood method based on the General Time Reversible model. A discrete Gamma distribution was used to model evolutionary rate differences among sites (5 categories, +G, parameter = 0.1000). The rate variation model allowed for some sites to be evolutionarily invariable ([+I], 9.76% sites). Bootstrap values (percentage of 1000 replicates) appear next to each corresponding branch. The tree was built with the Maximum Likelihood inference method, which was supported by the trees built with Maximum Parsimony, Neighbor Joining and Bayesian inference methods. * indicates luminescent strain.

An initial screening of phylogenetic analysis was performed with the 16S rRNA gene sequences of the pathogenic circulating strains and 362 *Vibrio* sequences reported in GenBank as pathogens and non-pathogens. The evolutionary history was inferred by using the Maximum Likelihood method based on the General Time Reversible model. A discrete Gamma distribution was used to model evolutionary rate differences among sites (5 categories, +G, parameter = 0.1000). The rate variation model allowed for some sites to be evolutionarily invariable ([+I], 9.76% sites). The pathogenic circulating strains of this study showed a greater similarity to the sequences of *Vibrio* reported as pathogens. To identify the isolates, a last analysis was performed using exclusively the 16S rRNA sequences obtained from GenBank that were more related to our sequences (Fig 1). Two groups of strains with high values of bootstrap support were identified (Fig 1). The first group contained eight sequences that presented similarity levels with sequences identified as *V*. *campbellii* and *V*. *harveyi* (Fig 1). All strains characterized in this study as luminescent belong to this group (Fig 1). A ninth luminescent strain was not included in the phylogenetic analysis, as the molecular identification was not possible with the obtained sequences (Fig 1). The second group included seven sequences of bacterial isolates with a high similarity level with identified sequences of *V*. *alginolyticus* and *Vibrio natriegens* (Fig 1). In addition, three different groups with low bootstrap values were also identified, which contained four sequences with similarities to *Vibrio inhibens*, *Vibrio owensii* and *Vibrio* sp. Strain PaH1.25 (Fig 1). In total, ninety-five percent of the isolated strains (19/20) belong to the *Harveyi* clade. Most of the strains were identified as *V*. *harveyi* and *V*. *alginolyticus* (12/19 strains). The least frequent strains were *V*. *campbelli*, *V*. *owensii*, *V*. *inhibens* and *V*. *natriegens* (Fig 1).

### Susceptibility of pathogenic *Vibrio* strains to antibiotics

All pathogenic strains were sensitive to furazolidone, ciprofloxacin, norfloxacin, nalidixic acid, chloramphenicol and florfenicol (Table 3). A total of 60, 95 and 90% of the strains were sensitive to tetracycline, fosfomycin and enrofloxacin (Table 3). All strains showed resistance or intermediate sensitivity to penicillin and oxytetracycline (Table 3). Few strains exhibited intermediate sensitivity to enrofloxacin (10%), oxytetracycline (15%) and tetracycline (10%) (Table 3). The complete list of diameters of the inhibition halos is provided in S2 Table.

All strains presented resistance to at least two antibiotics at the same time, being 50%, 45% and 5% of the strains resistant to 2, 3 and 4 antibiotics, respectively (Tables 3 and 4). The MAR index on average was 0.23 (Tables 3 and 4). The most prevalent pattern of multiple resistance was P-T (10/20 strains = prevalence of 50%, Table 4). The other most prevalent pattern was P-T-TE (7/20 strains = prevalence of 35%, Table 4). The other two MAR patterns were: P-T-Te (2/20 strains = prevalence of 10%, Table 4) and P-T-TE-FF (1/20 strains = prevalence of 5%, Table 4). All strains were resistant at the same time to penicillin and oxytetracycline (Tables 3 and 4). Forty percent (8/20) of the strains were resistant to both antibiotics of the tetracycline group (Tables 3 and 4).

**Table 3:** Multiple antibiotic resistance (MAR) and susceptibility of pathogenic circulating strains to antibiotics and commercial probiotics, through the results of the antibiogram tests.

Bacterial strain	Antibiotic susceptibility												Probiotic susceptibility				
	P	T	TE	FF	FUR	CIP	NOR	NAL	E	C	F	MAR index	P1	P2	P3	P4	P5
L15.25.1	R	R	R	S	S	S	S	S	S	S	S	0.27	R	R	S	R	I
L15.13.2	R	I	R	S	S	S	S	S	S	S	S	0.27	I	S	S	S	S
L15.31.1	R	R	S	S	S	S	S	S	S	S	S	0.18	R	R	S	S	S
L15.19.2	R	R	S	S	S	S	S	S	S	S	S	0.18	R	R	R	R	S
L15.26.1	R	R	S	S	S	S	S	S	S	S	S	0.18	S	S	R	R	S
L15.10.3	R	R	R	S	S	S	S	S	S	S	S	0.27	R	R	R	R	I
L15.29.1	R	R	R	R	S	S	S	S	S	S	S	0.36	R	R	R	R	S
L15.21.2	R	I	S	S	S	S	S	S	S	S	S	0.18	R	R	R	R	S
L15.5.2	R	R	R	S	S	S	S	S	S	S	S	0.27	S	R	R	R	S
L15.12.2	R	R	S	S	S	S	S	S	S	S	S	0.18	R	R	R	R	S
L15.19.1	R	R	I	S	S	S	S	S	S	S	S	0.27	R	R	R	R	S
L15.25.3	R	R	S	S	S	S	S	S	S	S	S	0.18	R	S	S	I	S
L15.26.3	R	R	R	S	S	S	S	S	S	S	S	0.27	I	R	R	R	S
L15.21.1	R	R	S	S	S	S	S	S	S	S	S	0.18	S	R	R	R	S
L15.11.2	R	R	S	S	S	S	S	S	I	S	S	0.27	R	R	I	I	S
L15.23.3	R	R	S	S	S	S	S	S	I	S	S	0.27	S	I	S	S	S
L15.23.1	R	R	S	S	S	S	S	S	S	S	S	0.18	R	R	R	R	S
L15.12.1	R	R	S	S	S	S	S	S	S	S	S	0.18	S	R	R	R	S
L15.10.4	R	R	I	S	S	S	S	S	S	S	S	0.27	R	R	R	R	I
L15.29.2	R	I	S	S	S	S	S	S	S	S	S	0.18	R	R	S	R	S

Antibiotics: penicillin (P), oxytetracycline (T), tetracycline (TE), fosfomycin (FF), furazolidone (FUR), ciprofloxacin (CIP), norfloxacin (NOR), nalidixic acid (NAL), enrofloxacin (E), chloramphenicol (C) and florfenicol (F). Probiotics: P1, P2, P3, P4 and P5. Susceptibility of the pathogenic circulating strains to antibiotics and probiotics are expressed as sensitive (S), intermediate (I) and resistant (R).

**Table 4:** Patterns of multiple antibiotic resistance (MAR) and MAR index.

Number of bacterial strains	MAR pattern	MAR index
10	P-T	0.18
7	P-T-TE	0.27
2	P-T-E	0.27
1	P-T-TE-FF	0.36

Penicillin (P), oxytetracycline (T), tetracycline (TE), enrofloxacin (E) and fosfomycin (FF).

Forty-five percent of the strains exhibited MIC values for oxytetracycline of less than 100 μg mL^-1^ (Table 5). Forty-five percent of the strains presented MIC values between 200–500 μg mL^-1^ (Table 5). Ten percent of the strains presented a high MIC value for oxytetracycline (> 3500 μg mL^-1^, Table 5). All strains presented sensitivity to florfenicol at low concentrations (≤ 40 μg mL^-1^, Table 5).

**Table 5:** Results of minimal inhibitory concentration (MIC) tests for authorized antibiotics for use in aquaculture, organic acids and essential oils on the growth of pathogenic circulating strains.

Bacterial strain	Minimal inhibitory concentration (μg mL^-1^)												
	Antibiotic		Organic acid									Essential oil	
	T	F	OA1	0A2	0A3	0A4	0A5	0A6	0A7	0A8	OA9	EO1	EO2
L15.12.1	500	5	>3500	>3500	>3500	2500	>3500	2500	2500	>3500	100	1000	3000
L15.12.2	500	8	>3500	>3500	>3500	200	>3500	2500	2500	>3500	300	1000	>3000
L15.5.2	>3500	2	>3500	>3500	>3500	2500	>3500	2500	2500	>3500	400	2000	>3000
L15.21.1	100	4	>3500	>3500	>3500	2500	>3500	2500	2500	>3500	400	2000	3000
L15.21.2	5	8	>3500	>3500	>3500	2500	>3500	2500	2500	>3500	400	900	3000
L15.23.1	20	10	>3500	>3500	>3500	2500	>3500	2500	2500	>3500	400	2000	>3000
L15.23.3	100	40	>3500	>3500	>3500	2500	>3500	2500	2500	>3500	400	2000	>3000
L15.25.1	>3500	4	>3500	>3500	>3500	2500	>3500	2500	2500	>3500	500	200	>3000
L15.25.3	250	40	>3500	>3500	>3500	2500	>3500	2500	2500	>3500	400	2000	>3000
L15.26.1	100	5	>3500	>3500	>3500	2500	>3500	2500	2500	>3500	200	2000	3000
L15.26.3	50	20	>3500	>3500	>3500	2500	>3500	2500	2500	>3500	100	2000	3000
L15.13.2	500	20	>3500	>3500	>3500	2500	>3500	1500	300	>3500	500	2000	>3000
L15.11.2	10	8	>3500	>3500	>3500	2500	>3500	200	2500	>3500	200	2000	>3000
L15.29.1	200	5	>3500	>3500	>3500	2500	>3500	500	2500	>3500	200	1000	>3000
L15.29.2	200	8	>3500	>3500	>3500	2500	>3500	1000	2500	>3500	2500	2000	>3000
L15.10.3	500	8	>3500	>3500	>3500	600	>3500	1000	300	>3500	300	2000	>3000
L15.10.4	5	2	>3500	>3500	>3500	1500	>3500	2500	2500	>3500	200	2000	>3000
L15.19.1	500	8	>3500	>3500	>3500	2500	>3500	2500	2500	>3500	200	2000	3000
L15.19.2	200	8	>3500	>3500	>3500	2500	>3500	2500	2500	>3500	200	3000	3000
L15.31.1	5	5	>3500	>3500	>3500	2500	>3500	2500	2500	>3500	100	3000	3000

Antibiotics: oxytetracycline (T) and florfenicol (F); Organic acids: OA1, OA2, OA3, OA4, OA5, OA6, OA7, OA8 and OA9; Essential oils: EO1 and EO2. Maximum concentration analyzed: 3500 (oxytetracycline and organic acids) and 3000 (essential oils) μg mL^-1^.

### Susceptibility of pathogenic *Vibrio* strains to probiotics

In general, the pathogenic strains exhibited low sensitivity to the tested probiotics, except to P5 (85% of sensitivity); 25, 15, 30, 5 and 15% of the strains were sensitive to P1, P2, P3 and P4, respectively (Table 3). Intermediate sensitivity was observed for 10, 5, 5, 10 and 15% of the strains to the probiotics P1, P2, P3, P4 and P5, respectively (Table 3). In terms of resistance, 65, 80, 65, 75 and 0% of the pathogenic strains were resistant to P1, P2, P3, P4 and P5, respectively (Table 3). Two isolates (L15.10.3 and L.15.10.4) were resistant or intermediate resistant to all tested probiotics (Table 3); strains L15.10.3 and L15.10.4 also presented the highest mortalities in the *P*. *vannamei* challenge test. The complete list of diameters of the inhibition halos is provided in S2 Table.

### Susceptibilities of pathogenic *Vibrio* strains to organic acids and essential oils

The MIC analyses showed that 100% of the strains were resistant to 5 products (OA1, OA2, OA3, OA5 and OA8) up to 3500 μg mL^-1^ of each product (Table 5), and 15, 25 and 10% of the strains were sensitive to OA4, OA6 and OA7, respectively, at concentrations equal to or lower than 1500 μg mL^-1^ (Table 5). In general, most of the strains showed sensitivity to product (OA9, Table 5). Product EO1 controlled 100% of the strains between 100 to 3000 μg mL^-1^. Forty percent of the strains (8/20) were sensitive up to 3000 μg mL^-1^ against EO2 (Table 5).

### Cell toxicity of selected products

The most efficient products in terms of bacterial sensitivity were organic acids OA6 and OA9, but OA9 was cytotoxic at all assayed concentrations, not showing significant differences of cell viability between the evaluated concentrations of OA9 (P = 0.802, Table 6). However, OA6 was not toxic for shrimp haemocytes between 100 and 400 μg mL^-1^, exhibiting similar values of cell viability at those levels (P ≥ 0.848, Table 6). Significantly lower levels of cell viability were reported for concentrations of OA6 from 1000 to 3000 μg mL^-1^ compared with concentrations from 100 to 400 μg mL^-1^ (P < 0.001, Table 6). No significant differences of cell viability were found at concentrations of OA6 from 1000 to 3000 μg mL^-1^ (P ≥ 0.129, Table 6). The haemocytes cell viability was not affected by florfenicol and oxytetracycline up to 2000 and 4000 μg mL^-1^, respectively (Table 6). Cell viability of all four control replicates was 100%.

**Table 6:** Cell viability (%) of *P*. *vannamei* shrimp haemocytes after exposure at varied concentrations of authorized antibiotics for use in aquaculture and the two most effective natural products against pathogenic circulating bacterial strains.

Concentration of products (μg mL^-1^)	Product			
	F	T	OA6	OA9
100	98.6 ± 4.7 ^d^	97.2 ± 2.7 ^b^	99.0 ± 1.5 ^c^	23.6 ± 2.4 ^a^
200	98.6 ± 5.0 ^d^	99.2 ± 0.9 ^b^	99.6 ± 0.6 ^c^	24.2 ± 3.4 ^a^
400	98.6 ± 3.4 ^d^	97.7 ± 2.7 ^b^	97.5 ± 2.5 ^c^	23.4 ± 3.1 ^a^
500	98.6 ± 5.3 ^d^	98.5 ± 2.4 ^b^	89.2 ± 2.6 ^b^	22.5 ± 1.5 ^a^
1000	96.1 ± 4.7 ^d^	98.7 ± 1.2 ^b^	26.4 ± 1.5 ^a^	21.9 ± 1.0 ^a^
2000	90.0 ± 3.1 ^d^	97.7 ± 4.3 ^b^	24.3 ± 2.0 ^a^	-
2500	72.0 ± 6.8 ^c^	97.7 ± 2.0 ^b^	22.1 ± 1.8 ^a^	-
3000	62.0 ± 3.7 ^bc^	92.7 ± 4.0 ^b^	22.7 ± 0.9 ^a^	-
4000	58.7 ± 4.7 ^b^	92.7 ± 3.1 ^b^	-	-
5000	44.3 ± 2.0 ^a^	84.3 ± 1.5 ^a^	-	-
6000	37.7 ± 1.5 ^a^	79.0 ± 1.0 ^a^	-	-
7000	37.3 ± 1.2 ^a^	76.3 ± 4.0 ^a^	-	-

Organic acids: OA6 and OA9; Antibiotics: florfenicol (F) and oxytetracycline (T). Means with different letters within a column indicate significant differences at P ≤ 0.05 by ANOVA and Tukey´s Honest Significant Difference tests.

## Discussion

The efficiency of therapeutic products is of vital importance for the control of aquaculture diseases. In the present study, we investigate through some *in vitro* analyses the antimicrobial effectiveness of antibiotics and commercially available therapeutic products used in Ecuador to control pathogenic bacterial strains of shrimp larvae. To perform these analyses, we isolated the strains that were circulating in the shrimp hatcheries, verified their virulence through challenge tests and identified their molecular similarity with previously reported pathogenic *Vibrio*. By doing this, we confirmed that we were working with the circulating strains that cause real problems at the production level. The results were dependent on the product, concentration of the product and bacterial strain.

Antibiotics were the most efficient therapeutic agents against the growth of pathogenic bacteria. Eight of the antibiotics inhibited the growth of all or most of the pathogenic bacterial strains, but most of these products are not authorized for use in aquaculture. We included several antibiotics in our evaluation because we wanted to investigate if the pathogenic circulating strains exhibited patterns of multiple antibiotic resistance, which could be associated with antimicrobial use [47]. In our study, the MAR index was on average 0.23, showing some level of resistance to antibiotics. High antibiotic resistance has been found in hatcheries worldwide, as well as higher MAR indexes in hatcheries rather than in shrimp farms [48, 49]. For instance, MAR index ranges from 0.21 to 0.38 have been reported for bacteria isolated from shrimp hatcheries [50, 49], whereas, MAR indexes in shrimp farms range from 0.11 and 0.32 [49–52]. The average MAR index determined in this study is low compared to values reported in other shrimp hatcheries. However, all strains were resistant at the same time to penicillin and oxytetracycline, both antibiotics used in human medicine. Similar observations of isolates with higher resistance to antibiotics used in human medicine than those used in aquaculture have been reported by several authors [53, 48]. Most of the sampled hatcheries are in a region of multiple anthropogenic activities, without wastewater treatment, which could be a source of antibiotic pollution.

In our screening, in addition to penicillin and tetracycline, oxytetracycline was a less efficient antibiotic, which is one of the two authorized antibiotics for use in aquaculture, and the antimicrobial most commonly used in Ecuador for shrimp larval stages, although now it is in decline. The MIC values found in our study are high and similar to those reported by the shrimp culture. For instance, an average MIC of 304 μg mL^-1^ has been reported for *Vibri*o isolated in Mexico [53], values up to 400 mg mL^-1^ for *Vibrio* isolated from water in hatcheries and farms in Brazil [50] and up to 512 μg mL^-1^ for *Vibrio* isolated from *Penaeus monodon* and *P*. *vannamei* shrimp in Thailand [54]. Nevertheless, oxytetracycline is toxic for *Penaeus stylirostris* larvae at concentrations from 135.5 to 238 μg mL^-1^ [55]. Fifty-five percent of our screened strains (11/20) presented MIC values for oxytetracycline in this toxic range or above, indicating that the application of this antibiotic is not suitable for most of the pathogenic circulating strains. Considering this observation, it cannot be discounted that the prolonged use of oxytetracycline may explain the observed resistance.

Florfenicol is the other authorized antibiotic for use in aquaculture and was highly efficient in controlling bacterial growth at low concentrations, with a MIC of 8 μg mL^-1^ for 75% of the strains. Our results are in accordance with the MIC values from 0.5 to 4 μg mL^-1^ found for *Vibrio* spp. isolated in Ecuador, USA, Japan and Thailand [56, 57] and values from 0.25 to 8 μg mL^-1^ for *Vibrio* spp. isolated in Mexico [53]. Florfenicol is a broad-spectrum antibiotic, with the same mechanism of action to that of chloramphenicol (inhibition of protein synthesis), and commonly used for the treatment of bacterial diseases in shrimp, such as necrotizing hepatopancreatitis [58]. Although it has been reported that florfenicol is toxic for *P*. *vannamei* larvae (Zoea 1) at concentrations higher than 20 μg mL^-1^ [59], we found that haemocytes could thrive up to 2000 ug mL^-1^ without any problem of cell unviability. Possibly, these seemingly contradictory results could be explained by the fact that MTT analyses were performed using haemocytes of juvenile shrimp, and at this stage, shrimp could handle a high concentration of this antibiotic. Sixteen of our screened strains (80%) showed MIC values equal to or higher than 5 μg mL^-1^, indicating a loss of bacterial sensitivity and a development of resistance to this antibiotic, and therefore not recommended as a control strategy at production level.

Given these conditions, it is therefore necessary for producers to consider alternative strategies for the control of pathogenic bacteria. In our study, only one commercial probiotic (P5) exhibited a high antagonistic capacity against the bacterial strains (85% of the strains). P5, whose declared composition is *V*. *alginolyticus*, has been employed as a probiotic in Ecuadorian hatcheries since 1992 [60, 61] and it has been observed that it enhances postlarvae survival and immune response when shrimp get to the juvenile stages [35]. The rest of the probiotics could inhibit the growth of 15–30% of the strains, which showed intermediate effects, and therefore could be considered as functional for the growth control of some pathogenic bacterial strains. Of the two strains that were not completely inhibited by probiotic P5, neither inhibited the other probiotics. The administration of either multiple or single probiotics remains controversial [62, 63], and, as regards this, although we did not test many probiotics, P5, a single strain, was the most efficient probiotic, whereas, P1, P2, P3 and P4 declared as mixture strains were not particularly effective.

Only one organic acid (OA9) showed inhibition of growth of most of the strains, at low concentrations. This product is a mixture of acetic acid, propionic acid and formic acid. These acids, as well as butyric acid, are efficient for the control of aquatic and shrimp pathogenic *Vibrio* [26, 28, 29]. OA6 was the second most efficient organic acid, and contains lactic, fumaric, citric, malic and succinic acids. Lactic and citric acids seemed to be the best organic acids to control pathogenic *V*. *harveyi* in *Macrobrachium rosenbergii* [64], but lactic acid can inhibit the pathogenic microbiota of fishes [65]. OA4 contains propionic acid and formic acid, whereas OA7 contains formic acid. Three of these four organic acids contain formic acid, which is considered to be particularly effective against pathogenic *Vibrio* [26]. In addition, OA9 contains three of the four organic acids reported as good bacterial inhibiters, including acetic acid, which is a good disinfectant of *V*. *parahaemolyticus* [66]. Possibly the five organic acids whose MIC were not determined, could be effective at higher concentrations than tested in this study, which makes them inefficient products.

Essential oils are effective for inhibiting bacterial growth; in our study the essential oil EO1, whose declared composition includes extract of oregano oil, efficiently inhibited the growth of all evaluated bacterial strains, with MIC values equal to or lower than 3 mg L^-1^. Similar results were observed by Teixeira et al. 2013 for other bacterial genera, where the *Origanum vulgare* essential oil was effective at MIC values lower than 5 mg mL^-1^ [67]. Possibly, the efficacy for the bacterial inhibition of EO1 might be related to the presence of thymol and carvacrol, two of the compounds of oregano essential oil [68, 69] that decrease the bacterial counts of *V*. *vulnificus*, *V*. *parahaemolyticus* and *V*. *cholera* in muscle and hepatopancreas of juvenile *P*. *vannamei* [70]. Carvacrol also increases the survival of *A*. *franciscana* larvae challenged with *V*. *harveyi* [71]. However, despite the high potential of the essential oil against bacterial growth, it could be toxic at high concentrations. Concentrations higher than 14.9 mg L^-1^ of carvacrol turned out to be toxic for *A*. *franciscana* [71], whereas concentrations of *O*. *vulgare* leaf crude extract and essential oil were toxic for both *A*. *salina* [72] and *P*. *vannamei* shrimp [44] at concentrations higher than 2 mg L^-1^ and 10 mg L^-1^, respectively. Other authors have mentioned the ability of essential oils to interrupt bacterial communication, decreasing bacterial virulence and pathogenicity [73, 74]. Therefore, it would be advisable to evaluate these properties of oregano essential oils and at the same time its toxic effect.

The tests performed in this work are designed to analyze whether the commercial products inhibit the growth or kill the pathogenic bacteria, but the natural products evaluated in this work could exhibit other modes of action not studied in this work. Therefore, further studies will be necessary to evaluate their efficacies, in terms of others mode of action, such as: capacity to disrupt bacterial communication, improvement of the shrimp immune system, colonization of the gastrointestinal tract, enhanced shrimp growth and survival, among others.

Ninety-five percent of the isolated strains (19/20) belong to the *Harveyi* clade, known to be the pathogenic clade for shrimp [75]. This was consistent with the results of the challenge tests, thus verifying that the isolated strains were pathogenic. Most of the strains were identified as *V*. *harveyi* and *V*. *alginolyticus* (12/20 strains), which have been a continual problem for the Ecuadorian hatcheries since 1988–1989 [2–4]. This study, however, has identified the fact that new pathogenic strains have appeared (*V*. *campbellii*, *V*. *owensii*, *V*. *inhibens* and *V*. *natriegens*) in the Ecuadorian hatcheries, diversifying the circulating bacteria and making it crucial to study the effectiveness of treatments for each pathogenic strain. Periodic surveys at regional level and further challenge tests could be performed to identify the pathogenic circulating bacterial strains and to focus on the bacteria of concern. At the same time, shrimp producers can apply relatively simple *in vitro* and *in vivo* analyses, such as those employed in this study and take adequate management decisions based on these results, which in turn could reduce the impact of bacterial diseases and increase profits.

## Supporting information

S1 Table This table contains the details of the products marketed in Ecuador as therapeutic agents against shrimp bacterial. (DOCX)

S2 Table This table contains the complete list of diameters of the inhibition halos obtained through antibiogram analysis for the antibiotics and probiotics evaluated in the study. (DOCX)

S3 Table This table contains the geographic coordinates of sample sites. (DOCX)
