# Supplementary material for: Efficacy assessment of commercially available natural products and antibiotics, commonly used for mitigation of pathogenic Vibrio outbreaks in Ecuadorian Penaeus (Litopenaeus) vannamei hatcheries
Source: PLoS One. 2019 Jan 30;14(1):e0210478. doi: 10.1371/journal.pone.0210478 (PMC6353134; doi:10.1371/journal.pone.0210478)
Supplement: S1 Table — (DOCX) [file pone.0210478.s001.docx]

**S1 Table.** Details of the products marketed in Ecuador as therapeutic agents against shrimp bacterial.

| **Product code** | **Product name** | **Company name** | **Lot number** |
| --- | --- | --- | --- |
| **P1** | EPICIN - G2 | EPICORE | 0620181 |
| **P2** | EPICIN – 3W | EPICORE | 0509181 |
| **P3** | Sanolife PRO-2 | INVE AQUACULTURE | 7117365908 |
| **P4** | Sanolife PRO-W | INVE AQUACULTURE | 7118254267 |
| **P5** | Cepa probiótica ILI | ESPOL | 272018 |
| **OA1** | ECOCITRO | ADILISA | 01M0/0809118 |
| **OA2** | Biotronic Top3 | BIOMIN | 37180153 |
| **OA3** | ADISALM | ADILISA | 02M0/0838318 |
| **OA4** | DRACID AQUA LD | BRENNTAG | No declared |
| **OA5** | Liptocitro | Agripac | 20180511028990 |
| **OA6** | ACID5FIVE | Distribuciones IR | EVSJG15TMAB |
| **OA7** | AQUAFORM | PROBAC | 242418 |
| **OA8** | FORMYCINE | FARMAVET | CT1732601N |
| **OA9** | FORDEX | CHEMICAL PHARM | 78229 |
| **EO1** | REGANO | RALCO NUTRITION | 8302017 |
| **EO2** | MIX OIL | AWP ANIMAL WELLNESS PRODUCTS | No declared |

Probiotics: P1, P2, P3, P4 and P5; Organic acids: OA1, OA2, OA3, OA4, OA5, OA6, OA7, OA8 and OA9; Essential oils: EO1 and EO2.
