# Supplementary material for: Efficacy assessment of commercially available natural products and antibiotics, commonly used for mitigation of pathogenic Vibrio outbreaks in Ecuadorian Penaeus (Litopenaeus) vannamei hatcheries
Source: PLoS One. 2019 Jan 30;14(1):e0210478. doi: 10.1371/journal.pone.0210478 (PMC6353134; doi:10.1371/journal.pone.0210478)
Supplement: S2 Table — (DOCX) [file pone.0210478.s002.docx]

**S2 Table.** Diameters (mm) of the inhibition halos for bacterial strains obtained through antibiograms for the evaluated antibiotics and probiotics.

| Bacterial strain | P | T | TE | FF | FUR | CIP | NOR | NAL | E | C | F | P1 | P2 | P3 | P4 | P5 |
| --- | --- | --- | --- | --- | --- | --- | --- | --- | --- | --- | --- | --- | --- | --- | --- | --- |
| L15.25.1 | 0 | 0 | 0 | 33 | 23 | 26 | 31 | 34 | 21 | 37 | 29 | 0 | 0 | 14 | 0 | 8 |
| L15.13.2 | 0 | 15 | 0 | 17 | 20 | 31 | 30 | 28 | 22 | 31 | 28 | 8 | 10 | 13 | 14 | 11 |
| L15.31.1 | 0 | 13.5 | 25 | 16 | 20 | 33 | 30 | 35 | 22 | 35 | 27 | 0 | 0 | 12 | 14 | 11 |
| L15.19.2 | 0 | 15 | 24 | 20 | 22 | 36 | 32 | 25 | 26 | 42 | 26.5 | 0 | 0 | 0 | 0 | 13.5 |
| L15.26.1 | 0 | 0 | 23 | 26 | 22 | 28 | 29 | 30 | 23 | 33 | 28 | 12 | 11 | 0 | 0 | 17 |
| L15.10.3 | 0 | 0 | 12.5 | 22 | 21 | 26 | 27 | 31 | 23 | 37 | 28 | 0 | 0 | 0 | 0 | 9 |
| L15.29.1 | 0 | 0 | 10 | 11 | 24 | 26 | 20.5 | 28 | 24.5 | 35 | 30 | 0 | 0 | 0 | 0 | 10.5 |
| L15.21.2 | 0 | 16 | 23 | 23.5 | 23.5 | 28 | 25.5 | 31 | 23.5 | 32 | 24.5 | 0 | 0 | 0 | 0 | 11.5 |
| L15.5.2 | 0 | 0 | 0 | 33 | 24 | 29 | 30 | 32 | 21.5 | 39 | 30 | 10 | 0 | 0 | 0 | 11 |
| L15.12.2 | 0 | 0 | 21 | 28 | 22 | 27 | 29 | 31 | 21 | 33 | 30 | 0 | 0 | 0 | 0 | 10.5 |
| L15.19.1 | 0 | 10 | 15 | 25 | 23 | 29 | 31 | 33 | 23.5 | 35 | 24.5 | 0 | 0 | 0 | 0 | 12.5 |
| L15.25.3 | 0 | 0 | 19.5 | 25 | 21 | 27 | 27 | 32 | 24.5 | 36 | 28 | 0 | 11 | 10 | 9 | 13 |
| L15.26.3 | 0 | 0 | 10.5 | 23.5 | 24 | 28 | 26.5 | 32 | 24.5 | 24 | 19.5 | 7 | 0 | 0 | 0 | 14.5 |
| L15.21.1 | 0 | 0 | 24.5 | 26 | 26.5 | 23 | 25 | 29.5 | 25 | 34 | 27.5 | 10 | 0 | 0 | 0 | 12.5 |
| L15.11.2 | 0 | 13.5 | 21 | 35 | 21.5 | 23 | 23 | 25 | 20 | 29 | 23.5 | 0 | 0 | 8 | 6 | 14.5 |
| L15.23.3 | 0 | 0 | 26 | 28 | 21 | 25 | 26 | 32 | 20 | 25 | 30 | 10 | 8 | 10 | 14 | 10 |
| L15.23.1 | 0 | 0 | 25 | 20 | 22 | 30 | 27 | 28 | 22 | 33 | 30 | 0 | 0 | 0 | 0 | 9.5 |
| L15.12.1 | 0 | 16.5 | 24 | 31 | 24.5 | 24 | 25.5 | 29 | 23 | 35 | 29 | 10 | 0 | 0 | 0 | 9.5 |
| L15.10.4 | 0 | 10 | 17 | 26 | 19 | 29 | 29 | 32 | 23 | 36.5 | 22 | 0 | 0 | 0 | 0 | 9 |
| L15.29.2 | 0 | 15 | 24 | 33 | 25 | 28 | 28 | 33 | 24 | 40 | 30 | 0 | 0 | 10 | 0 | 10.5 |

Antibiotics: penicillin (P), oxytetracycline (T), tetracycline (TE), fosfomycin (FF), furazolidone (FUR), ciprofloxacin (CIP), norfloxacin (NOR), nalidixic acid (NAL), enrofloxacin (E), chloramphenicol (C) and florfenicol (F). Probiotics: P1, P2, P3, P4 and P5.
