# Supplementary material for: Efficacy assessment of commercially available natural products and antibiotics, commonly used for mitigation of pathogenic Vibrio outbreaks in Ecuadorian Penaeus (Litopenaeus) vannamei hatcheries
Source: PLoS One. 2019 Jan 30;14(1):e0210478. doi: 10.1371/journal.pone.0210478 (PMC6353134; doi:10.1371/journal.pone.0210478)
Supplement: S3 Table — (DOCX) [file pone.0210478.s003.docx]

**S3 Table.** Locations and geographic coordinates of the sites where shrimp were collected

| **Site** | **Location** | **Geographic coordinate** |
| --- | --- | --- |
| 1 | Punta Carnero | -2,299748, -80,906985 |
| 2 | Mar Bravo | -2,245950, -80,949738 |
| 3 | Manglaralto | -2,121179, -80,757896 |
| 4 | Mar Bravo | -2,263682, -80,931337 |
| 5 | Mar Bravo | -2,243758, -80,952269 |
| 6 | Mar Bravo | -2,256498, -80,938006 |
| 7 | Mar Bravo | -2,242012, -80,954147 |
| 8 | Mar Bravo | -2,242501, -80,953546 |
| 9 | Mar Bravo | -2,244930, -80,951132 |
| 10 | Mar Bravo | -2,266744, -80,928334 |
